# CXCR4 Is Dispensable for T Cell Egress from Chronically Inflamed Skin via the Afferent Lymph

**DOI:** 10.1371/journal.pone.0095626

**Published:** 2014-04-21

**Authors:** Skye A. Geherin, R. Paul Wilson, Silke Jennrich, Gudrun F. Debes

**Affiliations:** Department of Pathobiology, University of Pennsylvania School of Veterinary Medicine, Philadelphia, Pennsylvania, United States of America; University of California, Riverside, United States of America

## Abstract

T cell recirculation through extralymphoid tissues is essential to immune surveillance, host defense and inflammation. In this process, T cells enter the tissue from the blood and subsequently leave via the afferent lymph. In the absence of inflammation, T cells require CCR7 expression to egress from the skin or lung, which is consistent with the constitutive expression of the CCR7 ligand CCL21 on lymphatic endothelium. However, during chronic inflammation alternative chemoattractants come into play, allowing *Ccr7*-deficient (*Ccr7^−/−^*) T cells to egress efficiently from affected skin. As T cell egress from inflamed sites is a potential control point of the inflammatory response, we aimed to determine alternative T cell exit receptors using a mouse and a sheep model. We show that CCR7^+^ and CCR7^–^ T cells exiting from the chronically inflamed skin were highly responsive to the CXCR4 ligand CXCL12, which was induced in the lymphatics in the inflamed site. Based on these findings, we hypothesized that CXCR4 mediates T cell egress from inflamed skin. However, pharmacological inhibition of CXCR4 did not affect the tissue egress of wildtype or *Ccr7^−/−^* CD4 and CD8 T cells after adoptive transfer into chronically inflamed skin. Similarly, adoptively transferred *Cxcr4^−/−^ Ccr7^−/−^* and *Ccr7^−/−^* T cells egressed from the inflamed skin equally well. Based on these data, we conclude that, while CXCR4 might play an essential role for other cell types that enter the afferent lymphatics, it is dispensable for T cell egress from the chronically inflamed skin.

## Introduction

T cells continuously recirculate through tissues providing immunosurveillance as well as effector functions during inflammation and infection. While naïve T cell preferentially recirculate between blood and lymphoid tissues, memory/effector T cells efficiently migrate into extralymphoid tissues and subsequently enter the afferent lymph to return to the blood via lymph nodes and efferent lymph [1], [2]. Mechanisms of T cell migration from the blood into tissues are key to the local inflammatory response and represent drug targets for inflammatory diseases and transplant rejection [3], [4]. Even though T cell egress from extralymphoid tissues is a potential therapeutic target to modulate inflammatory infiltrates, the underlying mechanisms of tissue exit are only poorly defined.

The endothelium of afferent lymph vessels constitutively expresses the CCR7 ligand CCL21 in many organs [5], [6]. We and others previously showed that CD4 and CD8 T cells require expression of the chemokine receptor CCR7 to egress via the afferent lymph from extralymphoid sites, such as skin, lung, and peritoneum [7], [8], [9]. Congruently, T cells accumulate in extralymphoid tissues in *Ccr7^−/−^* mice [10]. CCR7 is also a main guidance receptor for T cells to exit from inflamed tissue, which is reflected in drastically reduced cell egress when T cells lack *Ccr7* in models of acute inflammation, such as influenza A virus infection or early time points of adjuvant-induced skin inflammation [11], [12]. Moreover, antigen-recognition at the effector site decreases the exit capacity of CD8 effector T cells and correlates with reduced CCR7 function [11]. Thus, tissue exit represents a regulatory mechanism in inflammation that influences the quality of a tissue infiltrate. In addition, Mackay *et al.* recently showed that CD8 T cells that lack *Ccr7* show enhanced development into cutaneous tissue resident memory T cells (T_RM_ cells) [13]. These data suggest that down-regulation of the “T cell tissue exit program” contributes to the development of T_RM_ cells and protection against reinfection or control of persisting pathogens.

We recently found that the chronicity of inflammation determines the number of T cells leaving the skin through the afferent lymph and the molecules employed in the process. Specifically, chronic inflammation boosts the total number of T cells that egress from affected skin and allows T cells to exit in a CCR7-independent manner [12]. This CCR7-independent T cell exit from inflamed tissue is pertussis toxin sensitive and largely independent of S1P receptors, suggesting a requirement for alternative chemokine receptors [12].

The CXCR4 ligand CXCL12 is constitutively expressed in most organs [14], [15] and can also be found in lymphatic endothelial cells (LECs) in extralymphoid tissues [16], [17]. CXCL12 binds two receptors: CXCR4 and CXCR7. While CXCR4 is widely expressed by hematopoietic cells, including T cells, CXCR7 expression is largely restricted to non-hematopoietic cells [18], [19]. Deficiency in CXCL12 or CXCR4 is perinatally lethal due to alterations of neuronal and cardiovascular development [20], [21], [22], [23]. Most T cell subsets express CXCR4, and the CXCL12-CXCR4 axis operates in migration-related events, such as chemotaxis and triggering cell adhesion, but it also fulfills alternative functions, including cell survival, cell cycle progression, and T cell costimulation [24], [25], [26]. While CXCR4-CXCL12 functions in DC migration from inflamed skin to draining lymph nodes [27], it is currently unknown whether this receptor-ligand pair can mediate T cell egress from extralymphoid tissues.

In this paper, we found that (CCR7^+^ and CCR7^–^) T cells exiting from the chronically inflamed skin were highly responsive to CXCL12 and that CXCL12 was expressed by afferent lymphatics in the inflamed skin. These findings prompted us to hypothesize that the CXCR4- CXCL12 receptor-ligand pair mediates CCR7-independent T cell exit from the inflamed skin. However, neither pharmacological inhibition of CXCR4 nor genetic deficiency in *Cxcr4* decreased the tissue egress of *Ccr7^−/−^* CD4 and CD8 T cells, suggesting the contribution of alternative and/or redundant exit receptors.

## Materials and Methods

### Ethics Statement

All animal experiments were approved by the Institutional Animal Care and Use Committee of the University of Pennsylvania (protocol numbers 804337 and 804370). All surgical procedures in both sheep and mice were performed under aseptic conditions using isoflurane anesthesia, and all animals were treated with buprenorphine to prevent postoperative pain. Other methods to minimize suffering included the administration of buprenorphine to mice injected with Complete Freund’s Adjuvant (CFA).

### Animals, Skin Inflammation, and Surgical Methods (Lymph Cannulation in Sheep and Implantation of Osmotic Minipumps in Mice)

For experiments in sheep, mixed breed intact ewes or wethers, 5–10 months of age, were purchased from Animal Biotech Industries (Danboro, PA) or Pine Ridge Dorsets (East Berlin, PA). All mice used were on C57BL/6 backgrounds. CD45.1 congenic or CD45.2 C57BL/6 mice (The Jackson Laboratory) were used as wild-type controls and as recipients in adoptive transfer experiments. *Ccr7* ^−/−^ mice [28] were kindly provided by Eugene Butcher (Stanford University), crossed with *Cxcr4^+/−^* mice ([21]; The Jackson Laboratory) and bred in house.

To induce chronic (≥15-d old) skin inflammation in sheep, 0.3–0.5 ml CFA (Sigma-Aldrich) emulsified 1∶1 with sterile saline, was injected subcutaneously into the drainage area of the prefemoral (subiliac) lymph node, as described [12]. To elicit chronic (≥21 d) skin inflammation in mice, 10 µl of non-emulsified CFA was injected subcutaneously in the area of the hind footpads as described [12].

Pseudoafferent lymph vessels were generated in sheep by surgical removal of prefemoral lymph nodes and were cannulated as previously described [12], [29] Briefly, 6–12 weeks post-lymphectomy, pseudoafferent lymph vessels were surgically cannulated using heparin-coated catheters (Carmeda), and afferent lymph was continuously collected into sterile, heparinized (APP Pharmaceuticals) bottles.

To administer AMD3100 to mice, osmotic minipumps (model 1003D, ALZET Osmotic Pumps) were implanted in a surgical procedure. Sterile-filtered AMD3100 in PBS (Sigma-Aldrich) or PBS alone (Invitrogen) was loaded into the osmotic pumps before equilibration in sterile PBS at 37°C for 12 h according to the manufacturer’s instructions. Next, the pumps were implanted subcutaneously through a small skin incision between the shoulder blades. 12 h post surgery, these mice were used as recipients in adoptive transfers (see below).

### Fetal Liver Chimeras, Cell Isolation and Labeling, and Adoptive Transfer Exit Experiments

As a source of *Cxcr4^−/−^ Ccr7^−/−^* T cells, we generated *Cxcr4^−/−^ Ccr7^−/−^* fetal liver chimeras by mating *Cxcr4^+/−^ Ccr7^−/−^* mice. To generate control fetal liver chimeras, we also mated CD45.2^+^ *Ccr7^−/−^* mice and CD45.1 congenic wildtype mice. On day 13–14.5 of gestation, fetuses were harvested and fetal liver single cell suspensions prepared. The CXCR4 genotype of each fetal liver derived from *Cxcr4^+/−^ Ccr7^−/−^* matings was determined by PCR using conditions and the following primers provided by The Jackson Laboratory for strain B6.129X-*Cxcr4^tm1Qma^*/J; current stock number 004341: CXCR4-mutant (CAC GAG ACT AGT GAG ACG TG), CXCR4-WT (TTC TCA TCC TGG CCT TCA TC), CXCR4-forward (CTG TCA TCC CCC TGA CTG AT). 5- to 8-week-old CD45.1 wildtype mice served as recipients and were irradiated with a ^137^Cs source (Gammacell 40 Exactor, MDS Nordion) for 2 dosages of 5.5 Gy separated by 3–6 hours. Irradiated recipients were IV reconstituted with wildtype (CD45.1^+^), *Ccr7^−/−^* (CD45.2^+^), or *Cxcr4^−/−^ Ccr7^−/−^* (CD45.2^+^) fetal liver cells. ≥2 months post reconstitution, T cell chimerism was determined by analyzing multiple tissues of individual mice. CD4 T cell chimerism for *Ccr7^−/−^* and *Cxcr4^−/−^ Ccr7^−/−^* reconstituted mice was 58.1–74.4% and 51.5–62.8%, respectively. CD8 T cell chimerism for *Ccr7^−/−^* and *Cxcr4^−/−^ Ccr7^−/−^* recipients was 23.6–56.2% and 38.9–56.7%, respectively. Splenocytes from fetal liver chimeras were used as donor cells in adoptive transfer exit experiments.

For adoptive transfer exit experiments, lymphocytes were mechanically isolated from spleens with 40-µm cell strainer (BD Biosciences) and mononuclear cells further purified by gradient centrifugation with Histopaque-1083 (Sigma-Aldrich). Cells were incubated at 10^7^ cells/ml in HBSS with 25 mM HEPES and 0.2–0.4 µM CFSE (Molecular Probes) or 5 µM eFlour670 (eBioscience) for 5–10 minutes at 37°C. Both reactions were stopped by adding calf serum, and cells were washed 3-times with RPMI containing 5% serum and once in PBS prior to transfer. Fluorescent labels were alternated between experiments.

Adoptive transfer skin exit experiments were performed as described [7], [12]. Briefly, 9–15 week old recipient mice carried 3-week old CFA-induced skin inflammation in the hind footpads (elicited as described above). Donor cells were a mixture of fluorescently labeled wildtype and *Ccr7^−/−^* splenocytes from 4–12 month old donor mice when CXCR4 was inactivated through AMD3100. Alternatively, donor cells were a mixture of fluorescently labeled splenocytes from CD45.1 congenic wildtype, *Cxcr4^−/−^ Ccr7^−/−^*, and *Ccr7^−/−^* fetal liver chimeras. A total of 3–6×10^6^ CFSE- and eFluor670-labeled donor cells resuspended in 10 µl PBS were injected into the skin granuloma using a 10 µl Hamilton syringe (model 701N). 12 h after injection, the draining and non-draining (control) popliteal lymph nodes, as well as the spleens, of recipient mice were harvested and analyzed for transferred cells (based on fluorescent and/or congenic labels), and lymphocyte subsets were determined by flow cytometry. Aliquots of injected and migrated cells were quantified by flow cytometry using a bead standard (15 µm polystyrene beads; Polyscience Inc.). Cell counts were adjusted for differences in the input populations.

### Chemotaxis Assay

The assay was performed and analyzed as described [30]. Briefly, cells from the afferent lymph of sheep or mouse mononuclear splenocytes were incubated in RPMI containing 0.5% bovine serum albumin (Invitrogen) for 1 h. 5–10×10^5^ cells in 100 µl were added to 5-µm-pore-sized, 24-well tissue culture inserts (Corning costar). Recombinant mouse CCL21 (R&D Systems) and recombinant human or mouse CXCL12 (R&D Systems) were titrated in triplicates, and cells were allowed to migrate for 1.5 h. Lymphocytes in the migrated and input wells were quantified using a bead standard (15 µm polystyrene beads, Polyscience Inc.) combined with the flow cytometric analysis of T cell subsets.

To test the effect of AMD3100 on T cell chemotaxis, mouse splenocytes were incubated with 5 or 25 µM AMD3100 (Sigma-Aldrich) for 15–30 min in RPMI1640 with 0.5% BSA, before subjecting them to a chemotaxis assay in the presence of AMD3100.

### Flow Cytometry

Samples of ovine cells were preincubated with mouse and sheep IgG (Jackson ImmunoResearch) before labeling with fluorochrome-conjugated (FITC, PE, Alexa Fluor 700, Pacific Blue, Pacific Orange) monoclonal antibodies (mAbs). The following mouse anti-sheep mAbs were used: CD4 (clone 44.38; Serotec), CD8 (clone 38.65; Serotec), and γδ TCR (clone 86D; VMRD). Some antibodies were labeled prior to staining using Zenon labeling kits according to the manufacturer’s instructions (Invitrogen). For the staining of mouse cells, cells were preincubated with rat IgG (Jackson ImmunoResearch) and unconjugated anti-CD16/CD32 (clone 2.4G2; UCSF Monoclonal Antibody Core), before incubation with fluorochrome-conjugated (allophycocyanin PE, PE-Texas Red, PE-cyanine 5, PerCP-cyanine 5.5 PE-cyanine 7, Pacific Blue) mAbs. The following rat anti-mouse mAbs (all from Ebioscience) were used: CD4 (clone RM4–5), CD8 (clone 53–6.7), CD45RB (clone 16A), CD45.1 (clone A20), and CD45.2 (clone104). Samples were acquired on a BD LSRII using FACSDiva software (BD Biosciences) and analyzed with FlowJo software (Tree Star).

### Histology

For immunofluorescence staining, the mouse skin was fixed with 4% paraformaldehyde for 6 hours at 4°C, washed with Tris-buffered saline (TBS) (Sigma-Aldrich), and left overnight at 4°C in 30% sucrose (Fisher) diluted in TBS. Next, the skin was frozen in OCT (Sakura) and 6 µm sections prepared. Before staining, sections were rehydrated for 5–10 min at room temperature with 100 mM Tris-HCl (Teknova). Slides were blocked with unconjugated rat anti-mouse CD16/CD32 (clone 2.4G2; UCSF Monoclonal Antibody Core) and donkey or goat IgG (Jackson Immunoresearch) in TBS/0.05% Tween 20 (Sigma-Aldrich). Before incubation with the primary antibodies, a Streptavidin/Biotin Blocking Kit (Vector Labs) was used according to the manufacturer’s instructions. The slides were then incubated with polyclonal rabbit anti-mouse LYVE-1 (AngioBio) and goat anti-mouse CCL21 (R&D Systems) or monoclonal mouse anti-mouse CXCL12 (clone 79018; R&D Systems). Donkey anti-rabbit (H+L) biotin (Jackson Immunoresearch) and goat anti-mouse IgG1 FITC (clone RMG1–1; Biolegend) or donkey anti-goat Ig (H+L) FITC (Jackson Immunoresearch) were used as second step reagents, followed by streptavidin Texas Red (BD Biosciences), and 4′,6-diamidino-2-phenylindole (DAPI) (Invitrogen) costaining. To confirm specificity of the staining, appropriate isotype controls were used (data not shown). The slides were embedded with ProLong Gold Antifade (Invitrogen) and fluorescence images acquired on a Nikon Eclipse E600 microscope using a Photometrics CoolSNAP EZ camera and NIS-Elements BR 3.0 software.

### Statistical Analysis

All statistical analyses were calculated using GraphPad Prism software. Unless otherwise indicated, all values are reported as individual data points that represent individual animals and/or mean ± SD, and statistical significance was determined using the non-parametric Mann Whitney U or Wilcoxon matched pairs test.

## Results and Discussion

### Skin Exiting T Cells Chemotax toward CXCL12

CCR7 and other “exit receptors” that promote T cell egress from extralymphoid tissues reduce local T cell accumulation, and the function of such receptors is therefore relevant to the regulation of inflammation as well as regional host defense. As chronic skin inflammation augments the tissue egress of T cells and enables significant migration of *Ccr7*-deficient T cells through the afferent lymph [12], we aimed to determine which chemoattractant receptors besides CCR7 may mediate T cell egress from chronically inflamed tissues.

**Figure 1:**
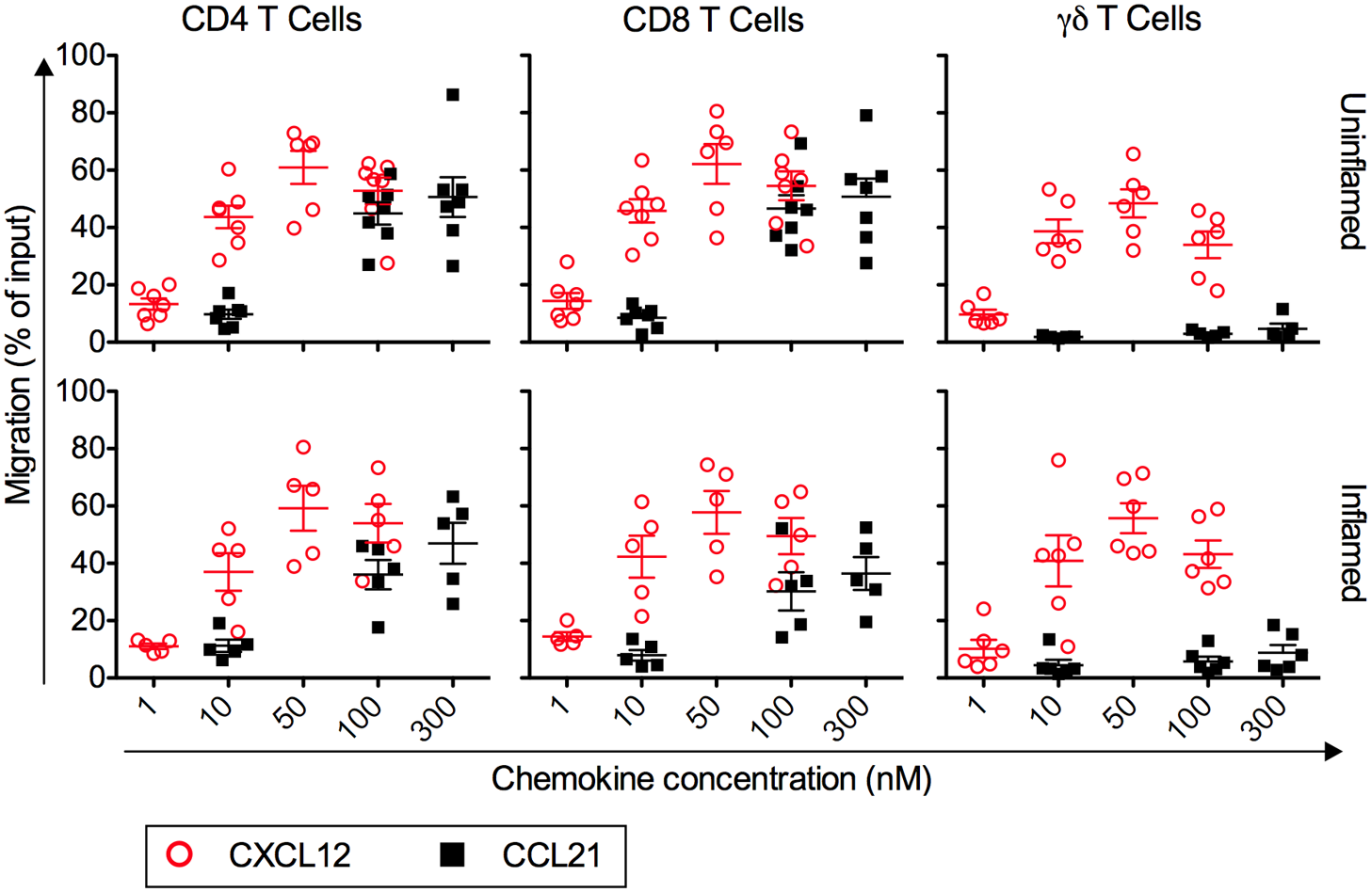
Ovine T cells that exit the inflamed and uninflamed skin efficiently migrate to CXCL12. Lymphocytes traveling in afferent lymph draining uninflamed or chronically inflamed skin (>d15 post induction of inflammation with CFA) were collected from sheep, and chemotaxis was tested toward mouse CCL21 and human CXCL12 in a Transwell chemotaxis assay. Migration of CD4, CD8 and γδ T cells is expressed as the percentage of each T cell subset that migrated to the lower chamber. Data points represent the mean migration of T cells from individual animals based on triplicate wells for each concentration, and the mean ± SEM for all animals analyzed is shown. N = 5–7 animals per condition and T cell subset.

Others reported a non-redundant role for CXCR4 in mediating DC migration from the skin to draining lymph nodes by mediating Langerhans cell migration from the epidermis to the CXCL12-expressing dermis [31] or by directing migration of dermal DCs into CXCL12 expressing lymph vessels [27]. Consequently, we wondered whether CXCR4 is a candidate receptor for mediating CCR7-independent T cell egress. In order to mediate exit, the receptor needs to be expressed by tissue exiting T cells, and its ligand must be present on LECs in the respective tissue. Because of a number of limitations, analysis of lymphocytes traveling in the afferent lymph for phenotypic and functional studies is not feasible in mice or humans. However, using a classic model of afferent lymph cannulation in the sheep [32], we were able to isolate tissue-exiting ovine T cells just after they had left the uninflamed skin and entered the afferent lymph. We also induced chronic (granulomatous) skin inflammation by subcutaneous injection of CFA and isolated lymph-borne T cells just after their egress from the site of inflammation [12]. Next, we compared the chemotactic responses of skin-exiting T cells toward CXCL12 and CCL21, ligands for CXCR4 and CCR7, respectively (Fig. 1). As we have recently described [30], inflamed and uninflamed skin-draining CD4 and CD8 T cells, but not co-isolated γδ T cells, chemotaxed toward CCL21 (Fig. 1). This data is consistent with CCR7 expression by lymph-borne CD4 and CD8 T cells and its absence on skin-recirculating γδ T cells [30], [33]. In contrast, all T cell subsets from the afferent lymph draining the uninflamed or inflamed skin, including CCL21-unresponsive γδ T cells, migrated efficiently (>50% of input on average) toward optimal concentrations of CXCL12 (Fig. 1). Thus, CXCR4 is a potential exit receptor for skin exiting T cells.

### CXCL12 is Expressed by Lecs in Chronically Inflamed Skin

**Figure 2:**
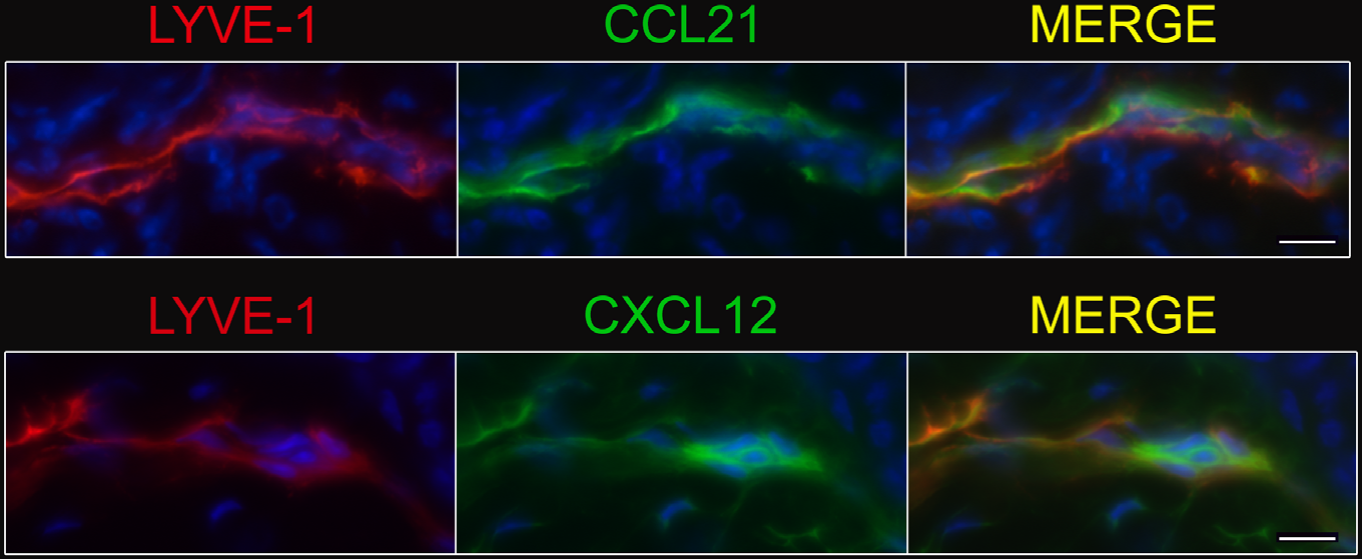
Afferent lymph vessels in chronically inflamed skin express CXCL12. Immunofluorescence staining of frozen sections of chronically inflamed skin (21 days after inflammation elicited by subcutaneous injection of CFA) for CXC12 and CCL21 by LYVE-1^+^ lymph vessels using DAPI counterstaining. One representative image of lymph vessels analyzed in 5 mice is shown. Scale bars, 10 µm.

Having determined that ovine skin exiting T cells in the skin-draining afferent lymph are highly responsive to CXCL12 (Fig. 1), we wondered whether cutaneous LECs expressed this chemokine, a prerequisite for CXCR4-driven T cell egress. While most studies find an upregulation of CCL21 in lymph vessels under inflammatory conditions (*e.g.* [11], [34], [35]), it is downregulated on the transcriptional level in cutaneous LECs in the oxazolone-challenge model of contact hypersensitivity [17]. Thus, it is possible that alternative chemokine receptor-ligand pairs, such as CXCR4-CXCL12 gain importance under inflammatory conditions. As there is significant CCR7-independent T cell exit from CFA-induced chronically inflamed mouse skin [12], we used this mouse model to assess expression of CCL21 and CXCL12 by LECs of the inflamed skin. We detected CCL21 by all LECs in the chronically inflamed skin using immunofluorescence staining of frozen sections (Fig. 2). Importantly, we could also detect clear CXCL12 expression by LECs in the inflamed skin with the brightest staining of LECs in close vicinity of hair follicles (Fig. 2). This finding is in line with data by others who found CXCL12 expression by lymphatics in inflamed tissues, such as the arthritic joint [16] and the skin in models of delayed-type hypersensitivity [17], [27]. We conclude that CXCL12 on LECs in chronically inflamed skin is well positioned to mediate the tissue exit of CCR7^+^ and CCR7^–^ CXCR4^+^ T cells.

### Pharmacological Inhibition of CXCR4 does not Impair T Cell Exit from Chronically Inflamed Skin

**Figure 3:**
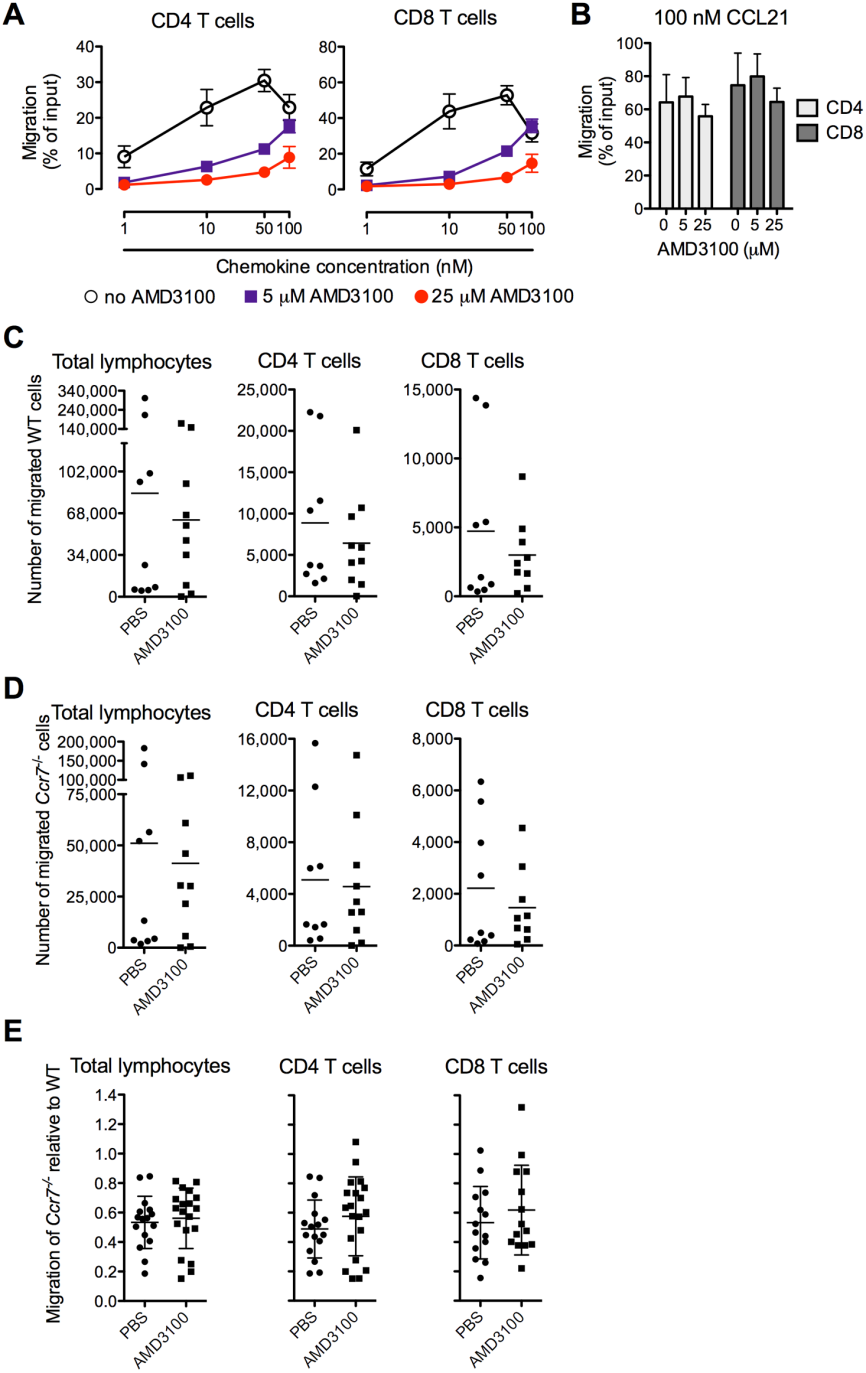
Pharmacological inhibition of CXCR4 does not impact the tissue exit of wildtype or Ccr7^−/−^ T cells. (**A–B**) Chemotaxis of mouse splenocytes was tested toward mouse CXCL12 (**A**) and CCL21 (**B**) in a Transwell chemotaxis assay in the presence or absence of CXCR4 inhibitor AMD3100 at indicated concentrations. Data represent mean ±SD of triplicate wells. One of two similar experiments is shown. (**C–E**) Mice carrying 3-week-old skin granulomas in the footpads were systemically treated with either PBS or 1000 µg/kg/h AMD3100 through subcutaneously implanted mini osmotic pumps. 12 h after implantation, a mixture of fluorescently labeled *Ccr7* ^−/−^ and wildtype splenocytes were transferred into the inflamed footpads. The numbers and phenotypes of cells that egressed from the skin and entered the draining lymph node were determined by flow cytometry 12 h after transfer. The numbers of wildtype (**C**) and *Ccr7* ^−/−^ (**D**) lymphocyte subsets that migrated to the draining lymph nodes are shown. Data points represent individually analyzed mice of groups of 7–10 recipient mice per group; horizontal lines indicate the mean of each group. One of two experiments with similar results (**C** and **D**) or both experiments combined (**E**) are shown. WT, wildtype.

As CXCL12 was expressed by LECs in chronically inflamed skin (Fig. 2) and CCR7^+^ and CCR7^–^ T cells exiting from such sites were CXCL12-responsive (Fig. 1), we hypothesized that CXCR4 acts as an exit receptor for T cells during chronic inflammation. We initially tested whether the selective CXCR4 antagonist AMD3100 (SID 791, JM3100, plerixafor; [36]) was able to block T cell chemotaxis to CXCL12 in an *ex vivo* chemotaxis assay. We found that AMD3100 blocked chemotaxis of splenic CD4 and CD8 T cells toward CXCL12, but not CCL21, in a concentration dependent manner (Fig. 3A and B). To test our hypothesis that CXCR4 is an essential T cell exit receptor in chronic inflammation, we used a mouse adoptive transfer model combined with pharmacological inhibition of CXCR4 using AMD3100. We induced skin granulomas in the footpads of mice by injection of CFA. We have previously shown that in this chronic inflammation model significant numbers of *Ccr7^−/−^* splenic and effector T cells egress from the inflamed skin after adoptive transfer [12]. Egress from the site of inflammation in this model likely requires additional chemoattractant receptors, such as CXCR4, because lymphocyte exit is sensitive to treatment with pertussis toxin [12], which irreversibly renders Gαi receptors unresponsive to ligand stimulation. Splenic T cells express CXCR4 and efficiently chemotax to its ligand CXCL12 (Fig. 3A; [37], [38]) and therefore were used as donor cells. Specifically, we transferred a mixture of fluorescently labeled wildtype and *Ccr7^−/−^* splenocytes into the site of inflammation on day 21 of the inflammatory response. We administered 1000 µg/kg/h of AMD3100 through the subcutaneous implantation of osmotic minipumps prior to cell transfer. 12 h after cell transfer, we analyzed the numbers and phenotypes of transferred cells that migrated from the inflamed skin to the draining popliteal lymph node in mice implanted with AMD3100- or PBS-loaded osmotic minipumps. Both wildtype and *Ccr7^−/−^* lymphocytes migrated into draining popliteal lymph nodes of AMD3100- and PBS-treated mice (Fig. 3C and D). In contrast, control tissues, i.e. spleens and contralateral lymph nodes, were devoid of transferred cells at this time point (data not shown), excluding migration via blood and confirming cell migration via the afferent lymph. There was no statistically significant difference in the numbers of migrated wildtype total lymphocytes, CD4 or CD8 T cells between AMD3100- and PBS-treated groups (Fig. 3C), suggesting that these cell subsets can resort to alternative tissue exit receptors, such as CCR7. However, AMD3100 treatment also did not impair the migration of *Ccr7* ^−/−^ total lymphocytes and CD4 or CD8 T cells, when comparing their migration in AMD3100- and PBS-treated mice (Fig. 3D). There was high variability in the numbers of migrated cells between individual recipient mice (Fig. 3C and D), and in the recipient mice with the highest numbers of donor cells recovered in the draining lymph node, B cells predominated among the migrated cells (data not shown). As such, we next analyzed the migration of *Ccr7* ^−/−^ cells relative to that of co-injected wildtype cells. This ratio-based quantification is independent of total cell numbers, as well as animal-to-animal variability, and allows for the combined analysis of multiple experiments, adding statistical power. We predicted that CXCR4 inhibition would more greatly affect *Ccr7* ^−/−^ T cells relative to wildtype, as the latter subset can resort to CCR7 for egress. We found that *Ccr7* ^−/−^ total lymphocytes as well as CD4 and CD8 T cells were reduced in draining lymph nodes by ∼50% on average compared with wildtype T cells in PBS-treated recipients (Fig. 3E). However, treatment with AMD3100, did not affect migration of *Ccr7* ^−/−^ total lymphocytes and CD4 and CD8 T cells relative to that of their wildtype counterparts (Fig. 3E). Our experiments employed a relatively high dosage (1000 µg/kg per hour) of AMD3100 to inhibit mouse CXCR4 *in vivo* similar to or higher than that successfully used by others [39], [40], and AMD3100 was able to block CD4 and CD8 T cell migration toward CXCL12 *ex vivo* (Fig. 3A). In addition, experiments employing the CXCR4 blocking peptide T22 [41] yielded similar results when used in adoptive transfer exit studies (data not shown). Together, the pharmacological CXCR4 blockade experiments suggest that this receptor is not required for T cell egress from the chronically inflamed skin.

It is unlikely that CXCL12 expressed on LECs in chronically inflamed skin was able to mediate T cell egress through its alternative receptor, CXCR7 [42], [43], as this receptor is a scavenging receptor for CXCL12 [44] that is not typically present in T cells [18], [45]. In addition, as AMD3100 is an antagonist of CXCR4 but not CXCR7 [46] that effectively blocked splenic CD4 and CD8 T cell chemotaxis to CXCL12 (Fig. 3A), CXCR7 can be excluded as a potential receptor mediating CXCL12-induced migration in our used T cell populations.

### Genetic Inactivation of Cxcr4 in Ccr7^−/−^ T Cells does not Further Impair Tissue Exit

**Figure 4:**
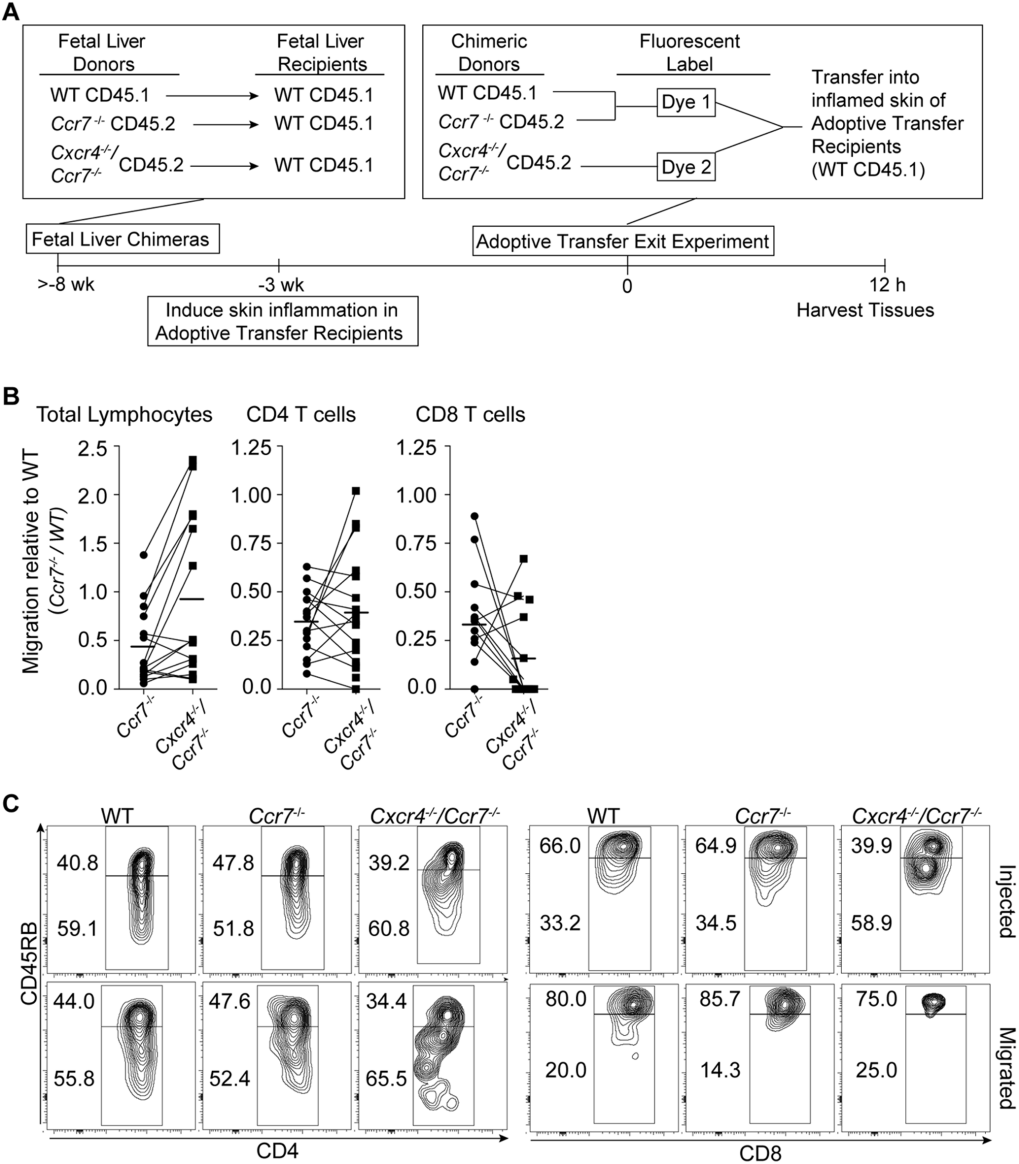
Cxcr4 deficiency does not reduce the tissue exit of Ccr7^−/−^ T cells from chronically inflamed skin. (**A–C**) Adoptive transfer exit experiments with fetal liver chimeric mice as a source of donor splenocytes. As depicted in the experimental scheme (**A**), fetal liver chimeras were generated by reconstituting lethally irradiated CD45.1^+^ wildtype recipient mice with fetal liver cells from (CD45.2^+^) *Cxcr4* ^−/−^ *Ccr*7^−/−^ or (CD45.2^+^) *Ccr7* ^−/−^ or (CD45.1^+^) wildtype donors. Splenocytes from the fetal liver chimeras were labeled with eFlour670 and CFSE, so that the combination of congenic maker and fluorescent label uniquely marked cells of each genotype. Labeled splenocytes of all three genotypes were mixed and co-injected into chronically inflamed footpads. (**B**) Analysis of migrated lymphocyte subsets recovered in the draining popliteal lymph nodes 12 h post cell transfer. Data is presented as the ratio of migrated to input cells for *Ccr7* ^−/−^ or *Cxcr4* ^−/−^ *Ccr*7^−/−^ relative to wildtype cells of each subset. Connected lines represent individually analyzed recipient mice. Horizontal, non-connecting lines indicate the mean of each group. (**C**) Flow cytometric analysis of memory (CD45RB^l^°) versus naïve (CD45RB^hi^) phenotype CD4 and CD8 T cells in injected and migrated populations for each genotype. Data combined from 2 experiments analyzing 5–10 recipient mice per experiment (**B**) or the cell phenotype of one out two experiment with similar results (**C**) is shown. WT, wildtype.

To address the role of CXCR4 as an essential T cell exit receptor, we employed a genetic approach to complement our experiments with pharmacological inhibitors targeting this receptor. In particular as the high animal-to-animal variability might have masked small drug effects (Fig. 3C and D), we sought an approach that allows for the analysis of competitive migration within individual mice. *Cxcr4* ^−/−^ T cells can still express CCR7, which is able to mediate egress through CCL21 found on LECs (Fig. 2). Therefore, we aimed to inactivate both CCR7 and CXCR4. As outlined in Figure 4A, we generated groups of fetal liver chimeras in CD45.1 congenic recipient mice through reconstitution with fetal liver cells from 3 different genetic backgrounds: wildtype (CD45.1^+^), *Cxcr4* ^−/−^ *Ccr7* ^−/−^ (CD45.2^+^), and *Ccr7* ^−/−^ (CD45.2^+^). ≥2 months after reconstitution, these fetal liver chimeras served as donors for adoptive transfer exit studies. We induced skin granulomas in CD45.1 congenic mice, and on day 21 of the inflammatory response, we transferred a mixture of CFSE-labeled CD45.1^+^ wildtype and CD45.2^+^ *Cxcr4* ^−/−^ *Ccr7* ^−/−^ and eFluor670-labeled CD45.2^+^ *Ccr7* ^−/−^ splenocytes into the inflamed footpads (Fig. 4A). The congenic markers, together with the fluorescent labels, allowed for the identification of the genotype of all transferred cell types and comparison of their migration in individual recipient mice (Fig. 4A). As in the previous experiments (Fig. 3), the draining popliteal lymph nodes and control sites (contralateral lymph nodes and spleens) were isolated and transferred cells enumerated and phenotyped. As expected, deficiency in *Ccr7* led to a reduction in tissue exit (migration from inflamed skin to draining popliteal lymph nodes) of total lymphocytes, CD4 and CD8 T cells relative to wildtype cells (Fig. 4B). Transferred cells in contralateral lymph nodes or spleen were below the level of detection (not shown), excluding migration from the blood into inflammation draining lymph nodes. Importantly, there was no statistically significant difference in the tissue egress of *Cxcr4* ^−/−^ *Ccr7* ^−/−^ CD4 and CD8 T cells relative to their *Ccr7* ^−/−^ counterparts (Fig. 4B), confirming our results using pharmacological blockade of CXCR4 (Fig. 3). In contrast, in many recipient mice, migration of *Cxcr4* ^−/−^ *Ccr7* ^−/−^ total lymphocytes exceeded that of *Ccr7* ^−/−^ lymphocytes (*P* = 0.0134), suggesting that some non-T cells (presumably B cells) use CXCR4 for their retention in the chronically inflamed tissue. This would be in accordance with the known role of the CXCR4-CXCL12 axis in retaining B cells and other cell types in the bone marrow [47].

To determine whether there was a relative preference to egress for naïve vs. memory T cells of each genotype, we examined the expression of the memory marker CD45RB, which is highly expressed by naïve T cells and downregulated following activation [48]. We found that naïve and memory phenotype CD4 T cells of all three genotypes migrated with the same efficiency, which was reflected in similar percentages of CD45RB^hi^ and CD45RB^l^° subsets in the input and corresponding migrated populations (Fig. 4C). Analysis of CD8 T cells showed that there was enrichment in naïve (CD45RB^hi^) relative to memory (CD45RB^l^°) cells in the migrated fractions for all three genotypes (Fig. 4C), similar as seen in CD4 T cell egress from the uninflamed skin [7]. Together, we conclude that CXCR4 is not required for CCR7-independent T cell exit from chronically inflamed skin and that *Cxcr4* ^−/−^ *Ccr7* ^−/−^ naïve and memory CD4 T cells are able to exit from the chronically inflamed skin.

Even if not required, it is still possible that the CXCR4-CXCL12 axis functions in lymphatic T cell egress in concert with CCR7 and other exit receptors. As CXCL12 is able to enhance responsiveness of naïve T cells to suboptimal concentrations of CCR7 ligands, thereby enhancing CCR7-dependent migration into lymph nodes via high endothelial lymph nodes [49], it might fulfill a similar function in inflamed tissue by facilitating CCR7-dependent T cell migration through LECs. In the skin, CXCL12 is expressed in various dermal and epidermal locations by several cell types, including blood endothelium, LECs and fibroblasts [50]. This expression profile combined with the diverse functions of CXCL12 on T cells may support opposing roles in the regulation of T cell accumulation in inflamed skin by influencing both cell accumulation (through enhancing retention, local survival and costimulation) and egress (by attraction into draining lymphatics).

As there is a great deal of redundancy in chemoattractants mediating T cell migration into sites of inflammation, there is likely redundancy in the chemoattractant-chemoattractant receptor pairs that ensure egress from such sites. Indeed, in addition to ligands for CCR7 and CXCR4, LECs in sites of inflammation express a wide array of inflammatory chemokines, including ligands for CCR1, CCR2, CCR6, CXCR1, CXCR2, CXCR3, CX3CR1 [17], [51], some of which may drive T cell egress in the absence of CCR7 and/or CXCR4. The chemoattractant sphingosine-1 phosphate (S1P) may also contribute to tissue exit [52]; however, our studies indicate that S1P receptors play only a minor role in T cell egress from chronically inflamed skin [12].

It is also possible that skin-recirculating, but not splenic, T cells depend on CXCR4 for their egress. However, CD4 and CD8 T cells in the skin draining afferent lymph respond to variety of inflammatory chemokines besides ligands for CCR7 and CXCR4 (Fig. 1), including ligands for CCR2, CCR4, CCR5, CCR6, CCR8, CCR10, and CXCR3 [30], [53]. While CCR7^–^ γδ T cells in the afferent lymph migrate to CXCL12 (Fig. 1), they also chemotax to the CCR6 ligand CCL20 [30]. Thus, skin-recirculating T cells are well equipped to respond to a variety of additional chemokines, which are inducible in LECs. Furthermore, use of AMD3100 to block CXCR4 in the sheep, while monitoring T cells subsets in the skin draining afferent lymph, had no effect on the egress of CD4 and CD8 T cells and yielded inconclusive results for γδ T cells (data not shown). Thus, it is unlikely that skin recirculating T cells depend on CXCR4 for their egress from inflamed skin.

In conclusion, we have shown that while the CXCR4 ligand CXCL12 is expressed by LECs in chronic cutaneous inflammation and skin-exiting T cells chemotax to CXCL12, CXCR4 is dispensable for CD4 and CD8 T cell exit from the chronically inflamed skin.

## References

[1] Mackay CR, Marston WL, Dudler L (1990) Naive and memory T cells show distinct pathways of lymphocyte recirculation. J Exp Med 171: 801–817. 2307933 10.1084/jem.171.3.801 PMC2187792

[2] Bromley SK, Yan S, Tomura M, Kanagawa O, Luster AD (2013) Recirculating memory T cells are a unique subset of CD4+ T cells with a distinct phenotype and migratory pattern. J Immunol 190: 970–976. 23255361 10.4049/jimmunol.1202805 PMC3618989

[3] Griffith JW, Luster AD (2013) Targeting cells in motion: migrating toward improved therapies. European Journal of Immunology 43: 1430–1435. 23580465 10.1002/eji.201243183 PMC3772080

[4] Bachelerie F, Ben-Baruch A, Burkhardt AM, Combadiere C, Farber JM, et al (2014) International Union of Pharmacology. LXXXIX. Update on the extended family of chemokine receptors and introducing a new nomenclature for atypical chemokine receptors. Pharmacol Rev 66: 1–79. 24218476 10.1124/pr.113.007724 PMC3880466

[5] Gunn MD, Tangemann K, Tam C, Cyster JG, Rosen SD, et al (1998) A chemokine expressed in lymphoid high endothelial venules promotes the adhesion and chemotaxis of naive T lymphocytes. Proc Natl Acad Sci U S A 95: 258–263. 9419363 10.1073/pnas.95.1.258 PMC18193

[6] Saeki H, Moore AM, Brown MJ, Hwang ST (1999) Cutting edge: secondary lymphoid-tissue chemokine (SLC) and CC chemokine receptor 7 (CCR7) participate in the emigration pathway of mature dendritic cells from the skin to regional lymph nodes. J Immunol 162: 2472–2475. 10072485

[7] Debes GF, Arnold CN, Young AJ, Krautwald S, Lipp M, et al (2005) Chemokine receptor CCR7 required for T lymphocyte exit from peripheral tissues. Nat Immunol 6: 889–894. 16116468 10.1038/ni1238 PMC2144916

[8] Bromley SK, Thomas SY, Luster AD (2005) Chemokine receptor CCR7 guides T cell exit from peripheral tissues and entry into afferent lymphatics. Nat Immunol 6: 895–901. 16116469 10.1038/ni1240

[9] Hopken UE, Winter S, Achtman AH, Kruger K, Lipp M (2010) CCR7 regulates lymphocyte egress and recirculation through body cavities. J Leukoc Biol 87: 671–682. 20028772 10.1189/jlb.0709505

[10] Hopken UE, Wengner AM, Loddenkemper C, Stein H, Heimesaat MM, et al (2007) CCR7 deficiency causes ectopic lymphoid neogenesis and disturbed mucosal tissue integrity. Blood 109: 886–895. 17018859 10.1182/blood-2006-03-013532

[11] Jennrich S, Lee MH, Lynn RC, Dewberry K, Debes GF (2012) Tissue exit: a novel control point in the accumulation of antigen-specific CD8 T cells in the influenza a virus-infected lung. J Virol 86: 3436–3445. 22278253 10.1128/JVI.07025-11 PMC3302526

[12] Brown MN, Fintushel SR, Lee MH, Jennrich S, Geherin SA, et al (2010) Chemoattractant receptors and lymphocyte egress from extralymphoid tissue: changing requirements during the course of inflammation. J Immunol 185: 4873–4882. 20833836 10.4049/jimmunol.1000676 PMC3327166

[13] Mackay LK, Rahimpour A, Ma JZ, Collins N, Stock AT, et al (2013) The developmental pathway for CD103(+)CD8(+) tissue-resident memory T cells of skin. Nat Immunol 14: 1294–1301. 24162776 10.1038/ni.2744

[14] Nagasawa T, Kikutani H, Kishimoto T (1994) Molecular cloning and structure of a pre-B-cell growth-stimulating factor. Proc Natl Acad Sci U S A 91: 2305–2309. 8134392 10.1073/pnas.91.6.2305 PMC43359

[15] Tashiro K, Tada H, Heilker R, Shirozu M, Nakano T, et al (1993) Signal sequence trap: a cloning strategy for secreted proteins and type I membrane proteins. Science 261: 600–603. 8342023 10.1126/science.8342023

[16] Burman A, Haworth O, Hardie DL, Amft EN, Siewert C, et al (2005) A chemokine-dependent stromal induction mechanism for aberrant lymphocyte accumulation and compromised lymphatic return in rheumatoid arthritis. J Immunol 174: 1693–1700. 15661933 10.4049/jimmunol.174.3.1693 PMC3121555

[17] Vigl B, Aebischer D, Nitschke M, Iolyeva M, Rothlin T, et al (2011) Tissue inflammation modulates gene expression of lymphatic endothelial cells and dendritic cell migration in a stimulus-dependent manner. Blood 118: 205–215. 21596851 10.1182/blood-2010-12-326447

[18] Berahovich RD, Zabel BA, Penfold ME, Lewen S, Wang Y, et al (2010) CXCR7 protein is not expressed on human or mouse leukocytes. J Immunol 185: 5130–5139. 20889540 10.4049/jimmunol.1001660

[19] Humpert ML, Tzouros M, Thelen S, Bignon A, Levoye A, et al (2012) Complementary methods provide evidence for the expression of CXCR7 on human B cells. Proteomics 12: 1938–1948. 22623068 10.1002/pmic.201100581

[20] Nagasawa T, Hirota S, Tachibana K, Takakura N, Nishikawa S, et al (1996) Defects of B-cell lymphopoiesis and bone-marrow myelopoiesis in mice lacking the CXC chemokine PBSF/SDF-1. Nature 382: 635–638. 8757135 10.1038/382635a0

[21] Ma Q, Jones D, Borghesani PR, Segal RA, Nagasawa T, et al (1998) Impaired B-lymphopoiesis, myelopoiesis, and derailed cerebellar neuron migration in CXCR4- and SDF-1-deficient mice. Proc Natl Acad Sci U S A 95: 9448–9453. 9689100 10.1073/pnas.95.16.9448 PMC21358

[22] Tachibana K, Hirota S, Iizasa H, Yoshida H, Kawabata K, et al (1998) The chemokine receptor CXCR4 is essential for vascularization of the gastrointestinal tract. Nature 393: 591–594. 9634237 10.1038/31261

[23] Zou YR, Kottmann AH, Kuroda M, Taniuchi I, Littman DR (1998) Function of the chemokine receptor CXCR4 in haematopoiesis and in cerebellar development. Nature 393: 595–599. 9634238 10.1038/31269

[24] Thelen M (2001) Dancing to the tune of chemokines. Nat Immunol 2: 129–134. 11175805 10.1038/84224

[25] Suzuki Y, Rahman M, Mitsuya H (2001) Diverse transcriptional response of CD4(+) T cells to stromal cell-derived factor (SDF)-1: cell survival promotion and priming effects of SDF-1 on CD4(+) T cells. J Immunol 167: 3064–3073. 11544290 10.4049/jimmunol.167.6.3064

[26] Nanki T, Lipsky PE (2000) Cutting edge: stromal cell-derived factor-1 is a costimulator for CD4+ T cell activation. J Immunol 164: 5010–5014. 10799853 10.4049/jimmunol.164.10.5010

[27] Kabashima K, Shiraishi N, Sugita K, Mori T, Onoue A, et al (2007) CXCL12-CXCR4 engagement is required for migration of cutaneous dendritic cells. Am J Pathol 171: 1249–1257. 17823289 10.2353/ajpath.2007.070225 PMC1988874

[28] Förster R, Schubel A, Breitfeld D, Kremmer E, Renner-Muller I, et al (1999) CCR7 coordinates the primary immune response by establishing functional microenvironments in secondary lymphoid organs. Cell 99: 23–33. 10520991 10.1016/s0092-8674(00)80059-8

[29] Young AJ, Hein WR, Hay JB (1997) Cannulation of lymphatic vessels and its use in the study of lymphocyte traffic. In: Levkovits I, editor. Manual of immunological methods: the comprehensive source book of techniques. San Diego: Academic Press. 2039–2059.

[30] Geherin SA, Lee MH, Wilson RP, Debes GF (2013) Ovine skin-recirculating gammadelta T cells express IFN-gamma and IL-17 and exit tissue independently of CCR7. Vet Immunol Immunopathol 155: 87–97. 23838472 10.1016/j.vetimm.2013.06.008 PMC3982400

[31] Ouwehand K, Santegoets SJ, Bruynzeel DP, Scheper RJ, de Gruijl TD, et al (2008) CXCL12 is essential for migration of activated Langerhans cells from epidermis to dermis. Eur J Immunol 38: 3050–3059. 18924211 10.1002/eji.200838384

[32] Lascelles AK, Morris B (1961) Surgical techniques for the collection of lymph from unanaesthetized sheep. Q J Exp Physiol Cogn Med Sci 46: 199–205. 13759245 10.1113/expphysiol.1961.sp001536

[33] Vrieling M, Santema W, Van Rhijn I, Rutten V, Koets A (2012) gammadelta T cell homing to skin and migration to skin-draining lymph nodes is CCR7 independent. J Immunol 188: 578–584. 22156593 10.4049/jimmunol.1101972

[34] Martin-Fontecha A, Sebastiani S, Hopken UE, Uguccioni M, Lipp M, et al (2003) Regulation of dendritic cell migration to the draining lymph node: impact on T lymphocyte traffic and priming. J Exp Med 198: 615–621. 12925677 10.1084/jem.20030448 PMC2194169

[35] Eberhard Y, Ortiz S, Ruiz Lascano A, Kuznitzky R, Serra HM (2004) Up-regulation of the chemokine CCL21 in the skin of subjects exposed to irritants. BMC Immunol 5: 7. 15109401 10.1186/1471-2172-5-7 PMC419342

[36] Bridger GJ, Skerlj RT, Thornton D, Padmanabhan S, Martellucci SA, et al (1995) Synthesis and structure-activity relationships of phenylenebis (methylene)-linked bis-tetraazamacrocycles that inhibit HIV replication. Effects of macrocyclic ring size and substituents on the aromatic linker. J Med Chem 38: 366–378. 7830280 10.1021/jm00002a019

[37] Scimone ML, Felbinger TW, Mazo IB, Stein JV, Von Andrian UH, et al (2004) CXCL12 mediates CCR7-independent homing of central memory cells, but not naive T cells, in peripheral lymph nodes. J Exp Med 199: 1113–1120. 15096537 10.1084/jem.20031645 PMC2211897

[38] Debes GF, Dahl ME, Mahiny AJ, Bonhagen K, Campbell DJ, et al (2006) Chemotactic responses of IL-4-, IL-10-, and IFN-gamma-producing CD4+ T cells depend on tissue origin and microbial stimulus. J Immunol 176: 557–566. 16365450 10.4049/jimmunol.176.1.557

[39] Matthys P, Hatse S, Vermeire K, Wuyts A, Bridger G, et al (2001) AMD3100, a potent and specific antagonist of the stromal cell-derived factor-1 chemokine receptor CXCR4, inhibits autoimmune joint inflammation in IFN-gamma receptor-deficient mice. J Immunol 167: 4686–4692. 11591799 10.4049/jimmunol.167.8.4686

[40] Broxmeyer HE, Orschell CM, Clapp DW, Hangoc G, Cooper S, et al (2005) Rapid mobilization of murine and human hematopoietic stem and progenitor cells with AMD3100, a CXCR4 antagonist. J Exp Med 201: 1307–1318. 15837815 10.1084/jem.20041385 PMC2213145

[41] Murakami T, Nakajima T, Koyanagi Y, Tachibana K, Fujii N, et al (1997) A small molecule CXCR4 inhibitor that blocks T cell line-tropic HIV-1 infection. J Exp Med 186: 1389–1393. 9334379 10.1084/jem.186.8.1389 PMC2199089

[42] Balabanian K, Lagane B, Infantino S, Chow KY, Harriague J, et al (2005) The chemokine SDF-1/CXCL12 binds to and signals through the orphan receptor RDC1 in T lymphocytes. J Biol Chem 280: 35760–35766. 16107333 10.1074/jbc.M508234200

[43] Burns JM, Summers BC, Wang Y, Melikian A, Berahovich R, et al (2006) A novel chemokine receptor for SDF-1 and I-TAC involved in cell survival, cell adhesion, and tumor development. J Exp Med 203: 2201–2213. 16940167 10.1084/jem.20052144 PMC2118398

[44] Naumann U, Cameroni E, Pruenster M, Mahabaleshwar H, Raz E, et al (2010) CXCR7 functions as a scavenger for CXCL12 and CXCL11. PLoS One 5: e9175. 20161793 10.1371/journal.pone.0009175 PMC2820091

[45] Infantino S, Moepps B, Thelen M (2006) Expression and regulation of the orphan receptor RDC1 and its putative ligand in human dendritic and B cells. J Immunol 176: 2197–2207. 16455976 10.4049/jimmunol.176.4.2197

[46] Kalatskaya I, Berchiche YA, Gravel S, Limberg BJ, Rosenbaum JS, et al (2009) AMD3100 is a CXCR7 ligand with allosteric agonist properties. Mol Pharmacol 75: 1240–1247. 19255243 10.1124/mol.108.053389

[47] Ma Q, Jones D, Springer TA (1999) The chemokine receptor CXCR4 is required for the retention of B lineage and granulocytic precursors within the bone marrow microenvironment. Immunity 10: 463–471. 10229189 10.1016/s1074-7613(00)80046-1

[48] Birkeland ML, Johnson P, Trowbridge IS, Pure E (1989) Changes in CD45 isoform expression accompany antigen-induced murine T- cell activation. Proc Natl Acad Sci U S A 86: 6734–6738. 2528147 10.1073/pnas.86.17.6734 PMC297920

[49] Bai Z, Hayasaka H, Kobayashi M, Li W, Guo Z, et al (2009) CXC chemokine ligand 12 promotes CCR7-dependent naive T cell trafficking to lymph nodes and Peyer’s patches. J Immunol 182: 1287–1295. 19155474 10.4049/jimmunol.182.3.1287

[50] Avniel S, Arik Z, Maly A, Sagie A, Basst HB, et al (2006) Involvement of the CXCL12/CXCR4 pathway in the recovery of skin following burns. J Invest Dermatol 126: 468–476. 16385346 10.1038/sj.jid.5700069

[51] Johnson LA, Jackson DG (2013) The chemokine CX3CL1 promotes trafficking of dendritic cells through inflamed lymphatics. J Cell Sci 126: 5259–5270. 24006262 10.1242/jcs.135343 PMC3828594

[52] Skon CN, Lee JY, Anderson KG, Masopust D, Hogquist KA, et al (2013) Transcriptional downregulation of S1pr1 is required for the establishment of resident memory CD8(+) T cells. Nat Immunol 14: 1285–1293. 24162775 10.1038/ni.2745 PMC3844557

[53] Geherin SA, Fintushel SR, Lee MH, Wilson RP, Patel RT, et al (2012) The skin, a novel niche for recirculating B cells. J Immunol 188: 6027–6035. 22561151 10.4049/jimmunol.1102639 PMC3370056

